# Impact of Different Saccharides on the In-Process Stability of a Protein Drug During Evaporative Drying: From Sessile Droplet Drying to Lab-Scale Spray Drying

**DOI:** 10.1007/s11095-023-03498-w

**Published:** 2023-04-03

**Authors:** Johanna Dieplinger, Joana T. Pinto, Michael Dekner, Gerald Brachtl, Amrit Paudel

**Affiliations:** 1 Research Center for Pharmaceutical Engineering GmbH, Graz, Austria; 2 Institute of Process and Particle Engineering, Technical University of Graz, Graz, Austria; 3 Takeda Manufacturing Austria AG, Vienna, Austria

**Keywords:** biopharmaceutical, miniaturized droplet drying, saccharide, spray drying, stabilization

## Abstract

**Objectives:**

Solid biopharmaceutical products can circumvent lower temperature storage and transport and increase remote access with lower carbon emissions and energy consumption. Saccharides are known stabilizers in a solid protein produced via lyophilization and spray drying (SD). Thus, it is essential to understand the interactions between saccharides and proteins and the stabilization mechanism.

**Methods:**

A miniaturized single droplet drying (MD) method was developed to understand how different saccharides stabilize proteins during drying. We applied our MD to different aqueous saccharide-protein systems and transferred our findings to SD.

**Results:**

The poly- and oligosaccharides tend to destabilize the protein during drying. The oligosaccharide, Hydroxypropyl β-cyclodextrin (HPβCD) shows high aggregation at a high saccharide-to-protein molar ratio (S/P ratio) during MD, and the finding is supported by nanoDSF results. The polysaccharide, Dextran (DEX) leads to larger particles, whereas HPBCD leads to smaller particles. Furthermore, DEX is not able to stabilize the protein at higher S/P ratios either. In contrast, the disaccharide Trehalose Dihydrate (TD) does not increase or induce protein aggregation during the drying of the formulation. It can preserve the protein’s secondary structure during drying, already at low concentrations.

**Conclusion:**

During the drying of S/P formulations containing the saccharides TD and DEX, the MD approach could anticipate the in-process (in) stability of protein X at laboratory-scale SD. In contrast, for the systems with HPβCD, the results obtained by SD were contradictory to MD. This underlines that depending on the drying operation, careful consideration needs to be applied to the selection of saccharides and their ratios.

**Supplementary Information:**

The online version contains supplementary material available at 10.1007/s11095-023-03498-w.

## Introduction

Dry powder of protein biopharmaceuticals is generally produced via lyophilization. In recent years, there has been increasing interest in using spray drying as an alternative process for developing dry powder biopharmaceuticals. This technique enables rapid drying of a protein-excipient solution atomized as droplets in a hot stream of air to produce dry powder particles. The benefit of spray drying is inherent to its single-step nature and rapid processing times, which could allow its application to the continuous manufacturing of biopharmaceuticals [1–3]. Compared to lyophilization, spray drying became a favoured alternative due to its lower energy consumption [4, 5]. Furthermore, the particle size distribution (PSD) of dry powders produced via spray drying shows good reproducibility, and produced particles are generally small in size, providing additional advantages of this technique from a drug delivery standpoint such as for inhalation [5, 6]. For the ProCept spray dryer used in this study, the size of particles generated using the different nozzle types can be between 1 – 350 µm [7]. Based on the authors experience with the bifluid nozzle, the average particle sizes obtained are between 1 µm (Division 10, Dv_10_) and 90 µm (Division 90, Dv_90_) [8, 9]. A great variety of excipient classes can aid in the protection of the protein during drying, i.e., surfactants, amino acids, saccharides, polymers, or other proteins [10–14]. The excipients of main interest in this work are saccharides which have been shown to stabilize proteins during freeze-drying based on their size and steric hindrance [15]. During spray drying of protein-saccharide formulations, the protein is trapped in a glassy saccharide matrix, which leads to protein stabilization [15–17]. Two main mechanisms about how saccharides can stabilize proteins during drying have been widely discussed and reported elsewhere, namely the water replacement theory and the vitrification theory [1, 16, 18–22]. Additionally, in a recent study, different saccharides were analyzed according to their ability to successfully stabilize bovine serum albumin (BSA) after spray drying [23]. In the past 20 years, the production of biopharmaceuticals by spray drying has greatly increased [10], yielding the first commercial products, e.g. inhalable insulin Exubera® in 2006. [24]. In 2015 the first aseptic biologic produced via spray drying, Raplixa®, was approved by the US-FDA [25, 26]. However, the development of the spray drying process requires the use of a considerable amount of material to obtain an adequate good powder yield for analysis. Due to the limited availability and costs of biopharmaceutical materials, during the early phase of formulation development, miniaturized screening workflows can enable the generation of a considerable amount of data with a limited amount of material. Different miniaturized screening approaches are existing and are being used for predicting particle formation during spray drying. Generally, this can be divided into single droplet or film casting experiments. For single droplet drying, four main approaches have been described in greater detail elsewhere [27–29]: free-falling droplet, levitation of droplet (acoustic or air flow), droplet hanging on a thin glass filament, or dispensed on a hydrophobic surface. In thin film preparation, a liquid is dispensed on a hot surface and dried inside an adequate container [30]. Thin film drying experiments were reported to be complementary to drying experiments of single droplets [30]. The advantages and disadvantages of the different mentioned droplet drying techniques are presented in Table I.

**Table I:** Summary of Positive and Negative Aspects of Acoustic Levitation, Film Casting, and Other Single Droplet Techniques

Miniature techniques	Advantages	Disadvantages
Acoustic levitation	- Monitoring of kinetics and droplet shape during the drying process [27] - Contactless drying minimizes unwanted change in droplet morphology - small amounts of material needed [31]	- Evaporation rate indirect [27] - Acoustic waves influence the drying process and droplet position [27] - Production of large particles & long drying times [31]
Film casting	- Information on evaporation rate, kinetic and thermodynamic powder stability [32]	- Solvent evaporation not representing droplet geometry [32]
Free-falling droplet	- Mimics the drying process as of a spray dryer [29]	- Individual droplet analysis difficult [27] - Drying process cannot be tracked consistently [29]
Pendant droplet drying	- Different parameters of interest can be measured concurrently [29] - Development of particle morphology can be monitored [27]	- Difficult to put droplet into desired position [29] - Filament interferes with droplet morphology and transfer of heat [29]
Sessile droplet drying	- Possible to track size and temperature of the droplet as well as morphology and crust formation [28]	- Interference of hydrophobic surface with droplet morphology and transfer of heat [28]

So far, there is a lack of systematic screening approaches for selecting excipients for the spray drying of biopharmaceuticals. Likewise, aware of this issue, Morgan *et al*., 2019, have developed a screening and selection of excipient approach for spray drying of viral vectors [31]. Their work aimed to confirm the conservation of particle morphology between screening (miniaturized drying approaches) and methods for production (spray drying) [31]. In our work, we aimed to develop a similar approach that would enable the simple and miniaturized screening of excipients by giving us valuable understanding of the aggregation behaviour of protein-saccharide formulations during spray drying at lab-scale. We based our work on the approach by Both *et al*., 2019, where a sessile droplet apparatus was used to study single droplet drying by dispensing the sample on a hydrophobic membrane. [33]. Our miniaturized approach developed at the small scale has proven to successfully relate to the drying behaviour at the lab scale for the di- and oligosaccharides TD and DEX used, but not for the polysaccharide HPβCD. The simple setup does not require levitation, can be easily replicated in other laboratories, requires only small sample amounts (µL), and can offer valuable insights into the spray drying process when applying TD and DEX as excipients in the formulations.

## Materials and Methods

### Materials

The formulation of a highly water-soluble protein (hereon referred to as ‘protein X’) presenting a molecular weight of  ~52 kDa was provided by Takeda (Vienna, Austria) and used in this study. The lyophilized formulation contained the protein of interest in a concentration  ≥80% w/w. Additionally, albumin, polyethylene glycol, polysorbate 80, and salts were also present. The disaccharide Trehalose Dihydrate (hereon referred to as ‘TD’) (Merck KGaA, Germany; Mw = 378.33 g/mol), the cyclic oligosaccharide Hydroxypropyl-β-cyclodextrin, Kleptose® HP ORAL GRADE (hereon referred to as ‘HPβCD’) (Roquette Frères, France; Mw = 1,501 g/mol) and the polysaccharide Dextran 40 EP (hereon referred to as ‘DEX’) (Pharmacosmos A/S, Denmark; Mw = 40,000 g/mol) were selected based on their different molecular weights, structures, and their known potential to stabilize proteins [14, 34–37]. They were used to prepare aqueous saccharide-protein solutions (further referred to as ‘S/P-formulations’) for miniaturized drying and spray drying (Table II). According to information about TD obtained from literature [34–37], 321:4 mM was chosen as the upper S/P molar ratio for using this disaccharide. For HPβCD, 61:4 mM was chosen as the upper S/P molar ratio based on available material. For DEX, 6:4 mM was chosen as the upper S/P molar ratio as this represented the maximum solubility with the protein in the formulation. The value of 4 mM of protein used in the formulation instead of 1 mM originates from the fact that 20% w/w was set as a fixed protein concentration for each formulation. Purified water (TKA Wasseraufbereitungssysteme GmbH, Germany) was used as a solvent. A BIO-RAD Gel Filtration Standard (Bio-Rad Laboratories Ges.m.b.H., Austria) was used for the relative quantification of monomer, dimer, and aggregate species by size exclusion chromatography (SEC).

**Table II:** Overview of Different Formulations and Their Composition of Protein X and Saccharides (Trehalose Dihydrate- TD, Hydroxypropyl-β-Cyclodextrin- HPβCD, and Dextran- DEX) in Water

Formulations	Saccharide class	Saccharide used	Mw saccharide [g/mol]	Saccharide concentration [mM]	S/P molar ratio [mM]
X_blank_MD	-	-	-	-	**0:1**
TD_X_HR_MD	Disaccharide	TD	378.33	321.13	**83.7: 1**
TD_X_MR_MD	Disaccharide	TD	378.33	79.99	**20.8: 1**
TD_X_LR_MD	Disaccharide	TD	378.33	20.04	**5.21:1**
HPβCD_X_HR_MD	Oligosaccharide	HPβCD	1,501.00	60.49	**15.7: 1**
HPβCD_X_MR_MD	Oligosaccharide	HPβCD	1,501.00	15.13	**3.93:1**
HPβCD_X_LR_MD	Oligosaccharide	HPβCD	1,501.00	0.49	**0.13:1**
DEX_X_HR_MD	Polysaccharide	DEX	40,000.00	6.00	**1.56:1**
DEX_X_MR_MD	Polysaccharide	DEX	40,000.00	1.28	**0.33:1**
DEX_X_LR_MD	Polysaccharide	DEX	40,000.00	0.64	**0.17:1**

3.84–3.85 mM is the fixed protein X concentration in each formulation (20%w/w). LR = low ratio, MR = medium ratio; HR = high ratio

### Methods

#### Sessile Single Droplet Drying (MD)

**Fig. 1:**
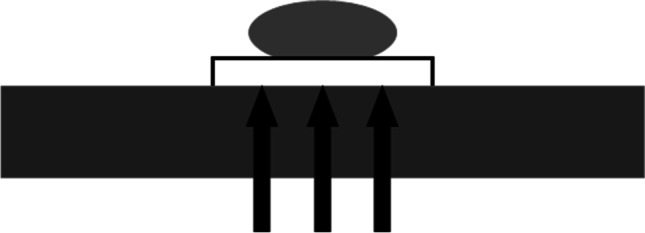
Schematic representation of the miniaturized method. The droplet is heated from the bottom by a hot plate (heat flow is indicated by black arrows).

Our developed miniaturized approach was a sessile single droplet drying method (referred to as the ‘MD’-method henceforth). For that, a droplet was dispensed onto a flat, hydrophobic surface (Fig. 1).

**Fig. 2:**
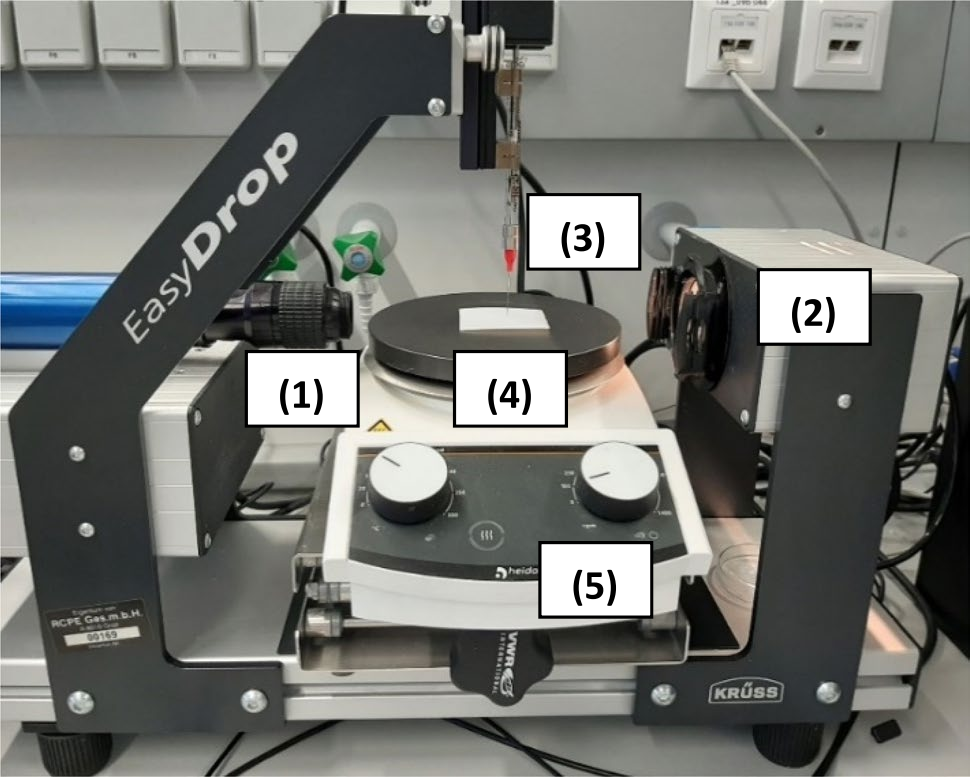
Overview of the Easydrop-setup developed for single droplet drying: (1) objective of camera, (2) light source, (3) Hamilton syringe, (4) Teflon-membrane with pp-film on top and (5) hot plate.

An EasyDrop equipment (EasyDrop, Krüss GmbH, Germany) with the software Drop Shape Analysis (DSA1 v1.92, Krüss GmbH, Germany) was used for following the drying process (Fig. 2). The magnification of 1 × zoom was applied during the measurements and a 1.8 mm tip was used to dispense single droplets with a volume of 15 μL onto a polypropylene-film, (pp-film) (Idena self-adhesive book film, 0.05 mm thickness), fixed to the top of a hydrophobic membrane (Teflon, roughed with K320 sandpaper) and placed on a hot plate (Hei-Standard magnetic stirrer, Heidolph, Germany) at a maximum hot plate temperature of 75 °C. This temperature was chosen based on the melting temperature (T_m_) of the protein X detected with Differential Scanning Calorimetry (DSF). The EasyDrop setup uses a halogen bulb as a light source and a monochrome interline CCD, 25/30 fps camera. The pictures of the single droplets were taken every minute for 15 min. The dried droplets were then removed from the pp-film and frozen until further analyses. The droplet drying was performed under standard room conditions (14.0—18.3 % RH and 20.5 – 22.1 °C). For dispensing the single droplets, Hamilton syringes with Krüss needles have been used. A timer was used to track the drying process over 15 min. A laser thermometer (Testo 845 Infrared Thermometer, US) with a measurement range of -35 to  +950 °C and a reference accuracy of up to  ± 0.75 °C was used to monitor the droplet temperatures during the process. The temperatures of the hot plate (start and end) and the pp-film (start and end) were measured as well.

### Analysis of Drying Rate, Shape, Volume, and Aspect Ratio of Evaporating Droplet

All presented figures were created using the OriginPro software, Version 2021 (OriginLab Corporation, Northampton, MA, USA).

The evaporation rate (drying rate) was calculated according to Eq. (1) [38]:

1 $${d}^{2}\left(t\right)={d}_{0}^{2}-kt$$

where $d$ is the diameter of the droplet over time, ${d}_{0}$ is the initial droplet diameter, $k$ is the evaporation rate, and $t$ is time. To this end, the radius $r$ of a spherical droplet having the same volume as the spherical cap over time was assumed and calculated according to Eq. (2):

2 $$r=\sqrt[3]{\frac{V}{4.19}}$$

To determine the evaporation rate, the radius of the droplet was plotted over time and the evaporation rate $k$ determined as the slope of the segment of the curve.

Assuming a spherical cap, which is the region of a sphere that lies above (or below) a given plane the droplet volume ($V$) and surface (*S*) was calculated according to Eqs. (3) and (4), respectively [39]:

3 $$V=\frac{1}{6}\pi h(3{a}^{2}+{h}^{2})$$

4 $$S=\pi ({a}^{2}+{h}^{2})$$

where, $h$ is the height and $a$ is the radius of the base circle. The height and the radius of the base were manually determined for all time points based on the pictures taken from the drop analysis software. Further the surface-to-volume ratio (S/V ratio) was calculated for each droplet, by simply dividing the value of the droplet surface after 15 min of drying by the value of the droplet volume after 15 min of drying. The aspect ratio (AR) was calculated for the dried particles using Eq. (5), where $w$ is the width and $h$ the corresponding height of a particle. Thus, the closer the value is to 1, the more spherical (or cubical) the particle is, values approaching zero indicate a needle shape.

5 $$AR=\frac{w}{h}$$

#### Spray Drying (SD)

The aqueous S-P formulations (20% w/w protein and different saccharide contents) were spray dried on a lab scale spray dryer (4M8-TriX, ProCepT, Belgium) equipped with a drying chamber of 1.4 m height. A 0.6 mm bi-fluid atomizing nozzle was used and the spray dryer was operated in open air loop. For the pump speed (20%; corresponded to 1.3–2.8 g/min), the air speed (0.8 m^3^/min), air inlet temperature (110 °C), nozzle pressure (0.6 bar) and cyclone airflow (180 L/min), the values were set and used as such for all spray drying experiments. The resulting outlet temperature (between 49.6 – 56.5 °C) was matched to the droplet temperature used during the MD and the powders were maintained for a maximum of 15 min inside the hot environment of the powder collector vessel in line with the timescales of the MD experiments. At the end of the experiments, the powders were stored in a fridge (2–8 °C) until further analysis.

#### Differential Scanning Fluorimetry (nanoDSF) of Feed Solution

The characterization of protein formulations in freshly prepared solutions was performed using a nanoDSF system (Prometheus NT. Plex, NanoTemper Technologies GmbH, Germany). The Tryptophan fluorescence of protein X was measured between 330 and 350 nm, providing the unfolding temperature, T_m_, of the protein, at which 50% of the protein is folded and 50% is unfolded. The temperature during the measurements was increased from 20 °C to 95 °C at a rate of 1 °C/min and 40% excitation power. For the sample preparation, 20 µL of each sample was pipetted into 384 well plates and centrifuged for 5 min at 5100 rpm (T = 20 °C). After centrifugation, the capillaries used for the analysis with the nanoDSF were filled with the samples through capillary forces, avoiding the formation of air bubbles. The measurements were performed in quintuplets *(n* = *5)*. The T_m_ values of the protein alone and in the presence of the selected excipients measured in the drying relevant solution were used as a stability descriptor for the comparison.

#### Size Exclusion Chromatography (SEC)

The protein samples were analysed via SEC *(n* = *3)* using an Agilent 1260 lnfinity HPLC-System (Agilent Technologies Inc., USA), equipped with a pre-column and a separation column. The software Empower™ 3 Feature Release 3 (Waters Corporation, USA) was used for data analysis. The phosphate buffer (pH = 7) was used as the mobile phase at a flow rate of 1 mL/min and the SEC column temperature was set to 20 °C to separate the protein sample by size. The injection volume of each sample was 25 μL and the concentration of the measured samples was 20 mg/mL. BIO-RAD Gel Filtration Standard (Bio-Rad Laboratories, Inc., USA) was used for the relative quantification of monomer, dimer, and aggregate species.

#### Analysis of Particle Size Distribution (PSD)

Laser Diffraction (HELOS/KR, Sympatec GmbH, Germany) was used to evaluate the particle size distribution of the protein powders produced by spray drying *(n* = *3)*. The optical mode R2 (for a size range of 0.45–87.5 μm) was chosen. The dry dispersion system (RODOS, Sympatec GmbH, Germany) was coupled to a vibrating drain (Vibri, Sympatec GmbH, Germany) to disperse the powder samples for analysis. A dispersing pressure of 2 bar was suitable to disperse the particles during a sampling time of 120 s. Windox5 Software (Sympatec GmbH, Germany) was used to analyse the volumetric particle size distributions. The S/V ratio was calculated by dividing SMD (Sauter Mean Diameter) by the VMD (Volume Mean Diameter). The diameters Dv_10_, Dv_50_ and Dv_90_ were used to calculate the span of the sample’s particle size distribution. Dv_10_, for example, describes the 10% of particles that are of that specific size in the volume distribution, Dv_50_ and Dv_90_ describe 50% and 90%, respectively. The distribution width or span was calculated according to Eq. (6):

6 $$span= \frac{{Dv}_{90}-{Dv}_{10}}{{Dv}_{50}}$$

#### Karl Fischer Titration (Moisture Content)

Determination of the water content of the spray dried protein powders (*n* = 3) was performed by Karl-Fischer titration (Titroline 7500 KF, SI Analytics, Germany) at conditions of 21.2 °C temperature and 25.7% humidity. Between 40 and 50 mg of powder (for a single measurement) were placed in the titrator cell. Using methanol (Aquastar® CombiMethanol, Merck KGaA, Germany), the water content was extracted for 1 h from the powders and was further quantified.

#### Wide Angle X-Ray Scattering (WAXS)

The WAXS analysis was performed for the original saccharide powders as well as the spray dried powders of protein and saccharide A high-flux laboratory camera (Hecus S3-Micropix, Austria) was equipped with a high-brilliance micro-beam delivery system and a point-focus optics (FOX3D), operated at 50W (1 mA and 50 kV) at an X-ray wavelength was 1.542 Å. A 1D-detector (PSD-50, Hecus X-ray Systems, Austria) was used to record the WAXS data within the angular range: between 17° < 2Ɵ < 27°. The spray dried powder samples were measured at room temperature and as singlets (*n* = *1*). The samples were transferred into glass capillaries (inner diameter of 2 mm) and exposed to the X-ray beam (diameter of 200 μm) for 600 s under constant rotation (9 rpm) to ensure the angular averaging of the scattering patterns of the powders.

## Results and Discussion

### Miniaturized Drying (MD)

When drying protein X in presence of different saccharides contained in the aqueous formulation, different performance of the saccharides used have been observed in terms of protein stability, evaporation rate, and resulting particle properties (volume, S/V ratio, and AR). The approximate S/P molar ratios are summarized in Table II (the same for MD and SD).

#### Impact of MD on Protein Stability

**Fig. 3:**
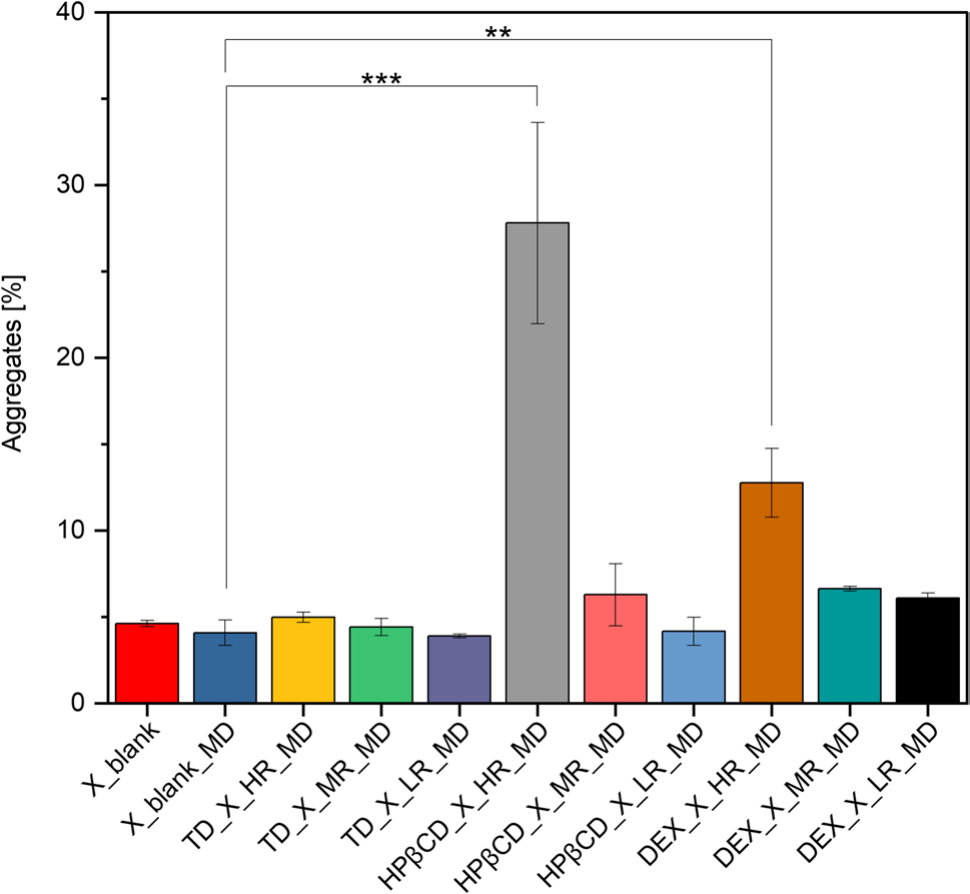
Statistical evaluation of the aggregates obtained by SEC-analysis (*n* = 3) of dried single droplets after MD (statistical differences are noted with asterisks: * *p* <  = 0.05, ** *p* <  = 0.01 and *** *p* <  = 0.001). The Bonferroni correction was applied.

The stability of protein X upon MD was examined using the relative content of protein aggregates and monomers determined via SEC-analysis. Interestingly, the drying of protein X formulation without saccharides, did not cause any statistically significant changes in terms of aggregate and monomer content of protein X (Fig. 3).

It was further observed, that adding TD in different ratios to the formulation with protein X, did not affect the aggregate and monomer content of the protein X. Strictly speaking, the percentage of aggregation in the presence of TD was very similar to when protein X was dried without the presence of any saccharide in the formulation. This revealed that the selected protein alone was stable during drying and no additional benefit in terms of stability was observed when the saccharides were added. In other words, the addition of TD did not induce aggregation and hence, does not lead to destabilization of the protein.

The situation with the use of HPβCD in the formulation with protein X, was different and complex. At HR, a significant destabilizing effect on the protein X was observed after MD, shown by increased aggregation and decreased monomer content (Fig. 3). Overall, the number of aggregates decreased and the amount of monomer increased when reducing the HPβCD-content in the formulation with protein X. This indicates that HPβCD might be inducing protein aggregation rather than reducing it. Looking into the existing literature, Serno *et al*., 2011, reported that β-CDs tend to bind to the exposed hydrophobic residue of protein (by including the part of aromatic amino-acids to the CD cavity) and act as molecular chaperone to stabilize the aggregation prone proteins [40]. Milani *et al*., 2020, found out that it depends on the ratio, in which HPβCD is used within the protein formulations, whether it will act as a lyoprotective saccharide or as a surface-active agent, the latter requiring lower amounts below 1% w/v. The authors found it to be most effective to use HPβCD in rather low weight ratios of 1:0.25 – 1:0.05 % for stabilization of IgG and further stated, that it is very difficult to assign a specific mechanism to a potential stabilization effect [41]. The inactivation of the enzyme β-galactosidase during spray drying, was reported to be prevented by adding HPβCD to the formulation [42]. Additionally, HPβCD stabilized IgG-based therapeutics during freeze-drying as well [43].

The same observations previously made with HPβCD, are true when DEX was used in its HR during MD. The fraction of aggregates decreased and the amount of monomer concurrently increased when the DEX-content was reduced in the formulation containing protein X. When comparing the specific S/P molar ratios, in which the three different saccharide classes have been used at, regarding their aggregation propensity, TD outperforms HPβCD, which in turn outperforms DEX. For the latter two, the stability of protein X was higher when using less amount in the formulations.

**Fig. 4:**
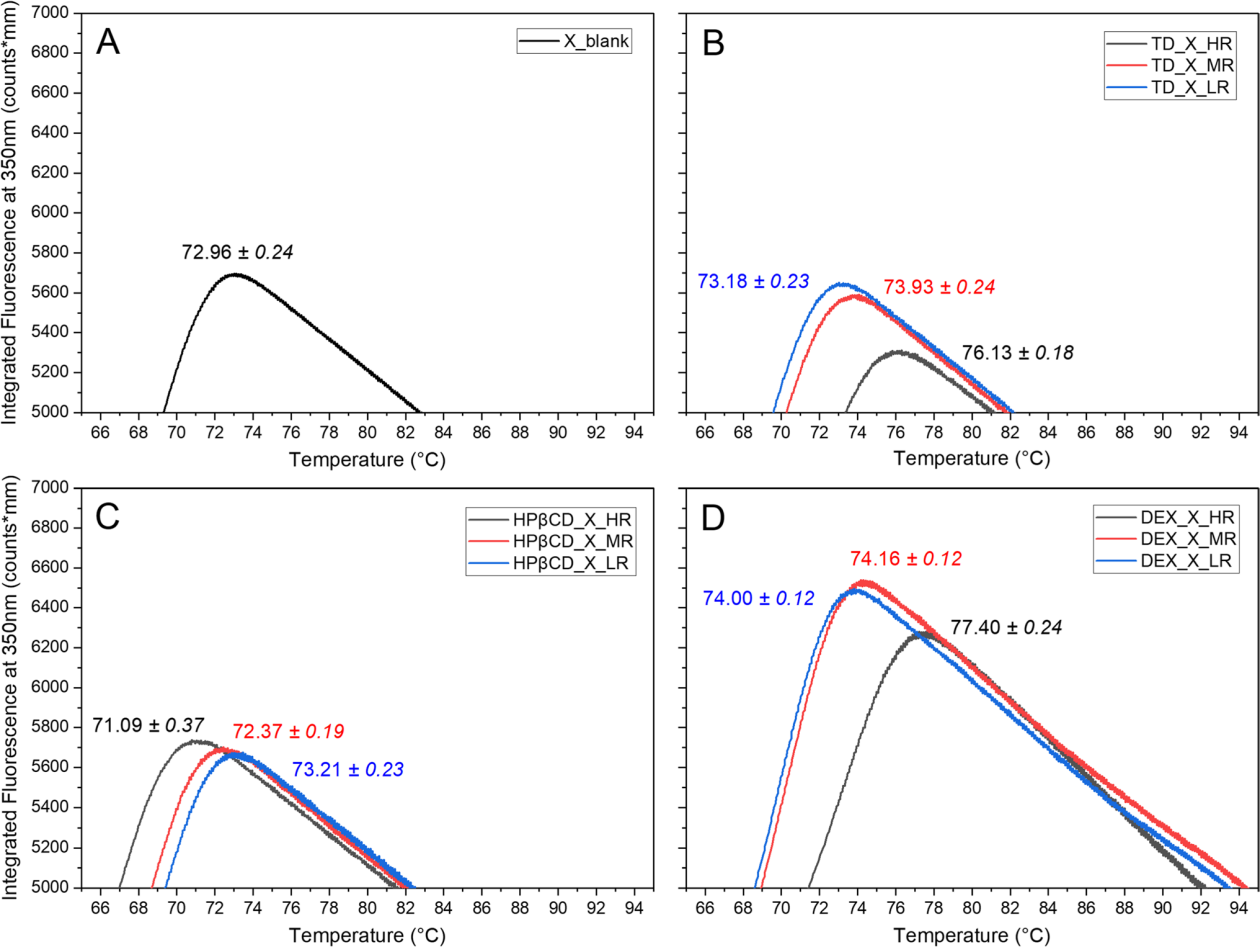
The first derivative of nanoDSF fluorescence spectra (*n* = 5) at 350 nm. The mean T_m_ ± standard error of the protein X with/without Saccharides present in the formulations are shown. For better visualization, only relevant regions are presented based on the maximum value of T_m_. Note: **A** represents the blank, containing protein X only., while **B** presents protein X with TD, **C** protein X with HPβCD and **D** protein X with DEX.

NanoDSF measurements were performed in order to shed more light on the observations made during MD. In nanoDSF measurements, the thermodynamic stability of proteins is analyzed by measuring the protein melting temperature (T_m_), while solutions of the biomolecule are heated up at a controlled rate. Therefore, the higher the T_m_ of a biomolecule, the more resistant it is to thermally induced unfolding [44–46]. In Fig. 4A, it can be seen that the T_m_-value for protein X alone is at 72.96 ± 0.24 °C. This value is quite similar to the values of TD, HPβCD and DEX used in their LR. Looking closer into the performance of each of the saccharides on the T_m_ shows that the addition of TD at MR and HR resulted in an increase in the T_m_ of protein X (Fig. 4B). This observation could indicate the beneficial effect of protecting protein X formulations from unfolding while heating its solutions.

For DEX, a similar stabilization trend was observed. Interestingly, the “temperature-window” of the thermal denaturation of protein for DEX samples during nanoDSF experiments seems about 10 °C broader (~69 – 94 °C) than for the other formulations tested (~67 – 83 °C), suggesting a slower denaturation. However, when looking at DEX in more detail, the observations made during MD and nanoDSF did not align and rather give inconsistent results. During MD, higher concentrations of this polysaccharide present in the formulations lead to destabilization of protein X, instead of stabilizing it. Interestingly, HPβCD tested at higher ratios, led to a decrease in T_m_ of protein X, indicating an induction of protein aggregation already in solution (Fig. 4C). This interpretation was reasoned by the fact that, if no saccharide was present in the formulation, the T_m_ was even higher than that of the formulation containing HR or MR of HPβCD. Indeed, this last observation with HPβCD matches with the MD-results, where the higher ratios of HPβCD were observed to detrimentally affect the stability of protein X formulations during drying (Fig. 3). Used in HR, HPβCD seems to destabilize the secondary structure of protein X, which aligns with the obtained DSF results.

As a possible explanation for the different observations between MD and nanoDSF we think, that the method applied in nanoDSF analysis might play a role. We propose that it might not be able to account for solute diffusion mechanisms, which occur during MD. Overall, T_m_ as the temperature of reversible unfolding, is not necessarily the same as T_agg_, that represents the temperature at which protein denaturation or aggregation starts. Hence, it makes sense that the DSF results obtained did not directly match with the aggregation content we have observed. The value for T_agg_ usually is lower than the value observed for T_m_, meaning, that the protein will start to aggregate before it will undergo unfolding at T_m_ in some cases [47]. Hence, the S/P formulation containing DEX at HR experienced aggregation already before unfolding at T_m_ during nanoDSF analysis. The next section will be dealing with discussions about how these mechanisms could have influenced the drying of the droplets and their resulting particle properties.

#### Impact of MD on Protein Particle Formation

**Fig. 5:**
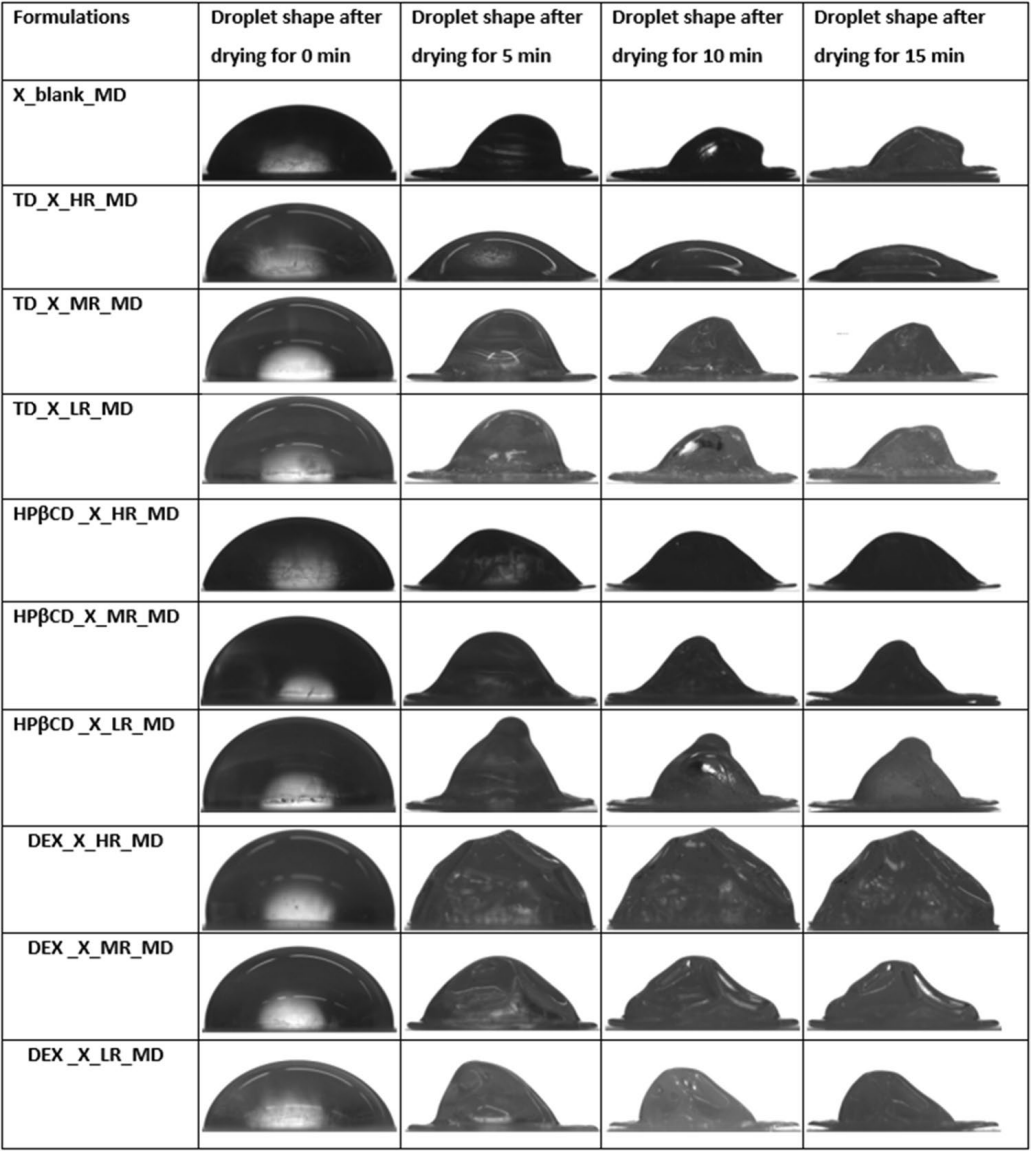
Droplet shapes of miniaturized single droplet drying experiments of formulations with different S/P molar ratios of TD, HPβCD or DEX and protein X. Protein X without saccharides is presented as blank.

The previously described miniaturized setup was used to monitor the particle formation when drying single droplet of formulations containing protein X and different saccharides. Based on generated droplet pictures, the evaporation rates, AR and S/V ratios were derived. Figure 5 presents different droplet shapes observed while performing MD.

**Fig. 6:**
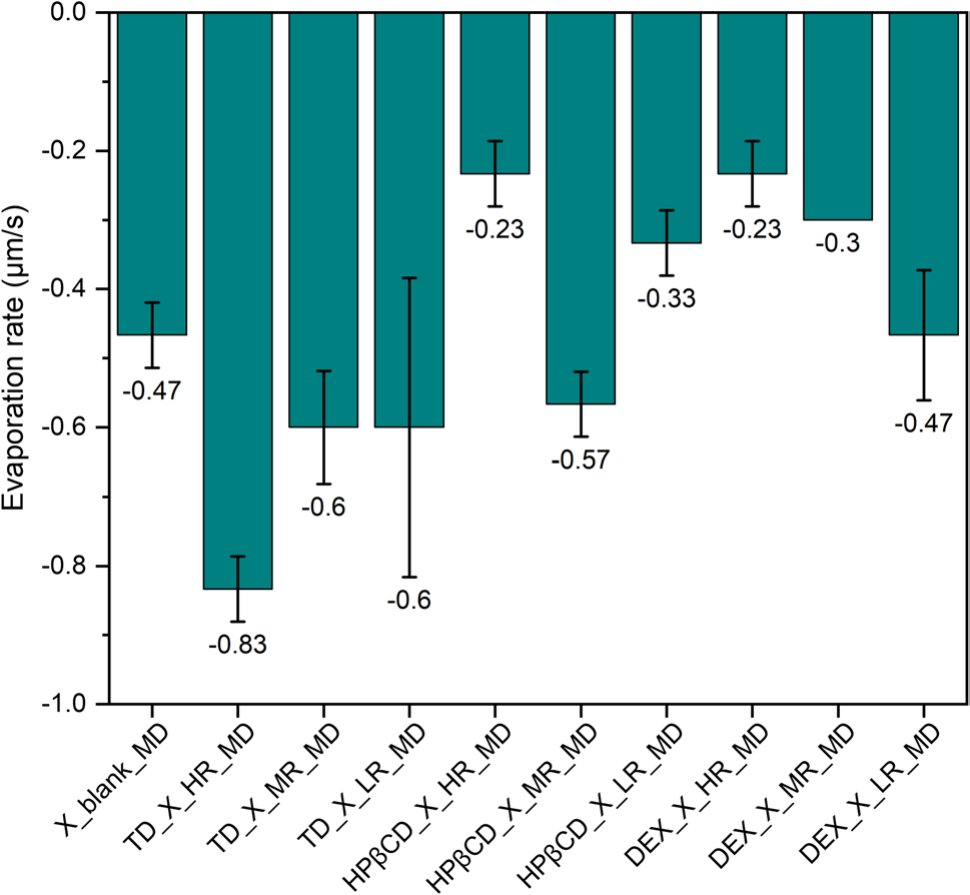
Different evaporation rates (µm/s) derived from measurements of dried droplets produced by miniaturized drying experiments are shown. Presented values are mean values and corresponding standard deviations are shown as error bars.

**Fig. 7:**
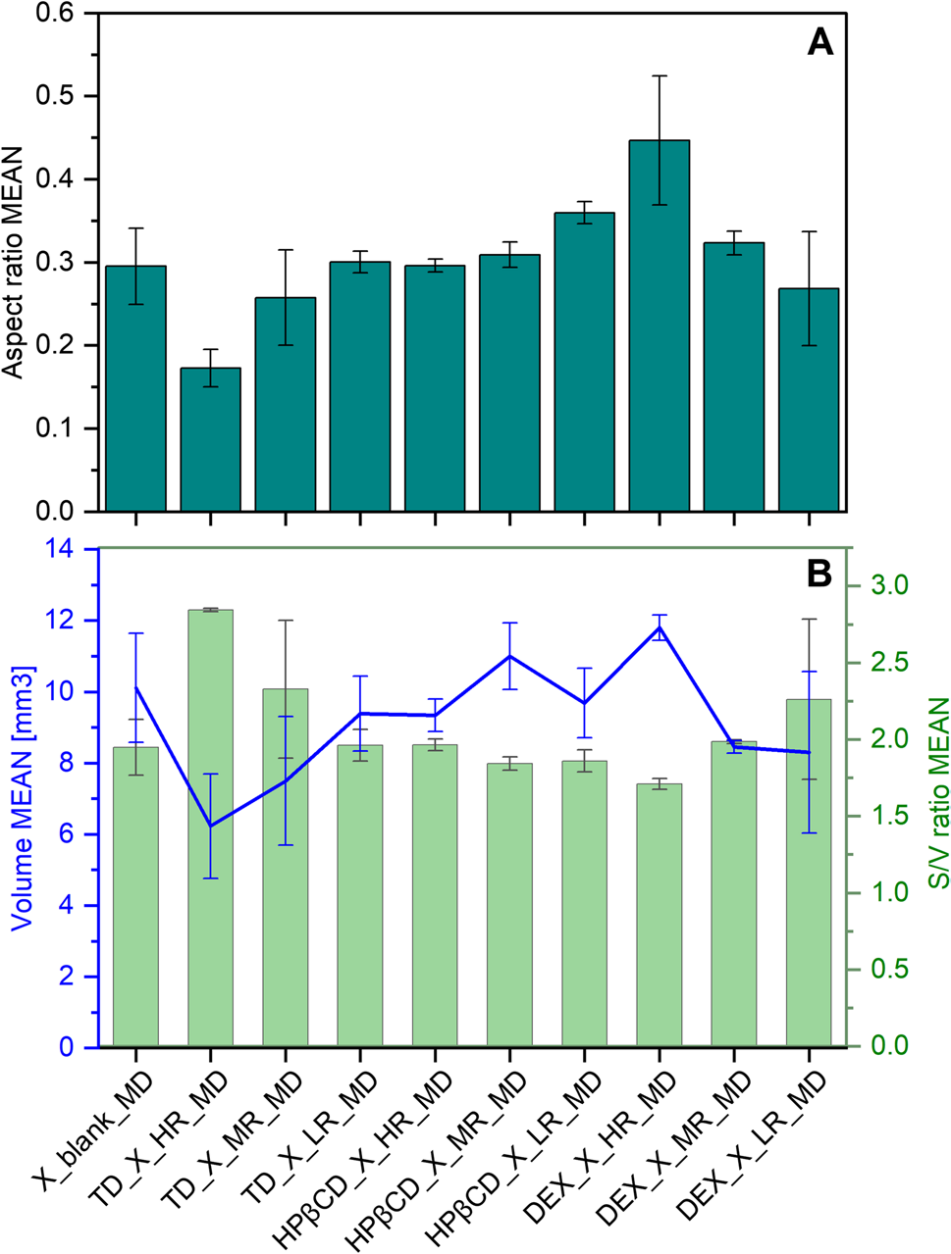
The AR (**A**) and the droplet volume (mm3) with AR (**B**) of droplets dried by MD are presented. Presented values are mean values and corresponding standard deviations are shown as error bars.

As can be seen, the drying of protein X solution without any saccharides results in dried particles with a “hat-shape” morphology (Fig. 5) [48]. This morphology is a consequence of the underlying mechanism and rate of the diffusion of solutes during water evaporation from the drying sessile drop. In such a MD, a pinned droplet is dried. As the drying proceeds, the temperature from the contact surface will be diffused throughout the whole droplet – from the bottom (the pinned contact area) to the top – and the droplet will be dried as a consequence. These established temperature gradients in sessile drops will be the driving force, next to the Marangoni effect, for solute diffusion from bottom to the top of the droplets [49]. In the case a crust has already formed, the solvent will diffuse out of the drying droplet’s crust, leaving a “hat-shaped” droplet behind [48], which appeared to be observed in this work. The Marangoni effect represents an interfacial flow, which works to restore the equilibrium of the solute concentrations at the interface of air and liquid, leading to circulation of the solute inside the drying droplet. This Marangoni flow is caused by differences in surface tension and can be reduced by surface-active agents [29]. Adding different saccharides to the formulation containing protein X, led to different evaporation rates during droplet drying, naturally affecting the dried particle’s morphology (Fig. 6). Protein X without the addition of any saccharide had an AR around 0.30 ± 0.05 (Fig. 7A). Our observations, that the pinned droplets first dry at the edges due to inhomogeneous evaporation and as a consequence, show a hat-shape morphology, align with Shao *et al*., 2021 [49] and Larson *et al*., 2014 [48]. According to the authors, the Marangoni effect could lead to increased velocities and could direct the particles to the droplet centre, where they will then accumulate and hence are supposed to create the typical hat-shape [48].

In the case of TD, adding more TD to the protein X formulation, led to a slightly faster evaporation (Fig. 6) than protein X alone. This is a natural phenomenon, as less water is present when more solids are added to the solution. The evaporation rate for TD used in HR compared to the evaporation rate when drying protein X only, decreases around 40% for the latter. Furthermore, the particle shapes of TD used in MR and LR as well as their evaporation rates, show very similar values to the protein dried without saccharides. Furthermore, the presence of more TD leads to dried particles with a very low AR (Fig. 7) suggesting a flat droplet shape, as the typical “hat-shape” morphology of protein X was lost with increasing TD content. Instead, particles with rather oblong shape were obtained, resulting in a higher S/V ratio and a lower AR. We assume that this also was caused by the higher solid content present in the droplet. During evaporation, the amount of solvent will decrease, leading to a high concentration of solids. A resulting viscous slurry will retard the solute diffusion [49]. Hence, the solutes cannot migrate anymore from the bottom to the top of the droplet and the “hat-shape” morphology is lost. Due to its smaller molecular size, TD might be able to stabilize protein X better than the much larger saccharides HPβCD and DEX, as steric hindrance will not be a problem for smaller molecules like TD to establish functional interactions with protein molecules for the stabilization. Nevertheless, none of the mentioned changes in droplet drying dynamics influenced the protein stability significantly.

**Fig. 8:**
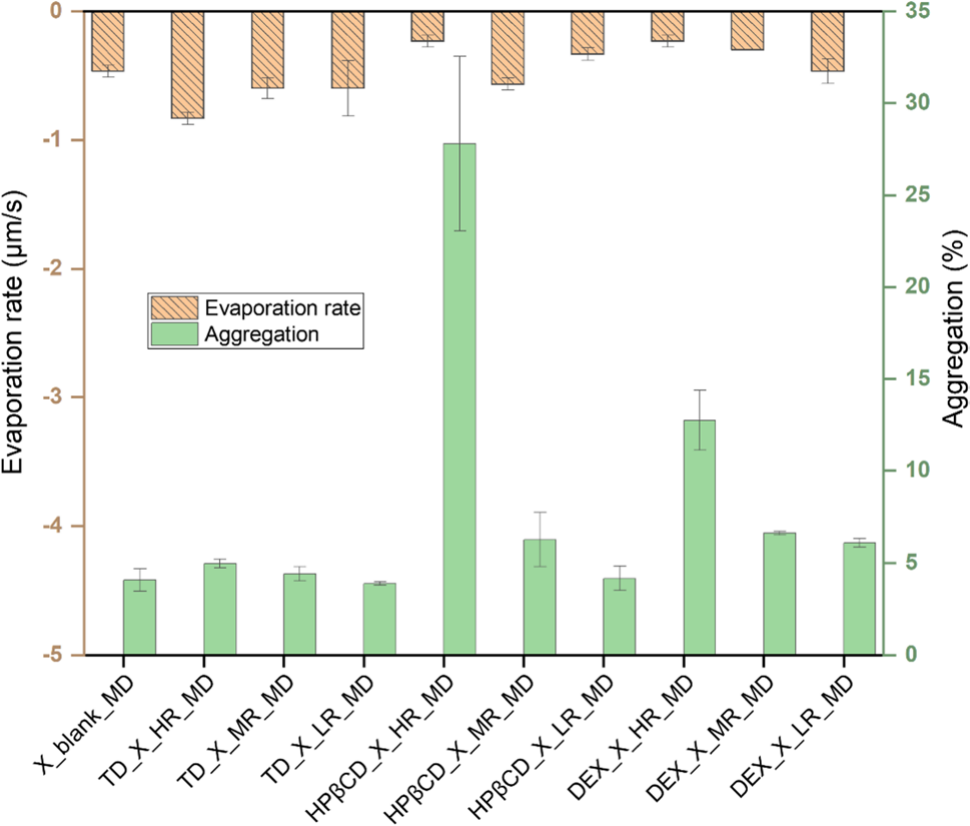
Comparison of evaporation rates (µm/s) and aggregation of different formulations examined during MD. Presented values are mean values and corresponding standard deviations are shown as error bars.

In the case of HPβCD addition, the increasing concentrations also led to the disappearance of the “hat-shaped” morphology of the protein particles, to an increase in S/V ratio and a decrease in AR, as was observed for TD. However, with HPβCD, surprisingly, a higher evaporation rate was observed when used at MR. Using HPβCD at HR, a notably lower evaporation rate was observed. This is a very significant and vital observation, given that the presence of HPβCD showed to destabilize the protein X when analysed with nanoDSF (Fig. 4C). Furthermore, the slower the evaporation rate, the more time the protein X will be spending in solution with HPβCD, leading to higher aggregation observed for HPβCD at its HR (Fig. 8).

In the case of DEX, very voluminous, uniform and large particles were obtained, when this polysaccharide was added to the formulation of protein X (Fig. 5). These particles showed a low S/V ratio and a high AR and the evaporation rate during drying was slower, if DEX was present in lower concentrations. This was indeed not surprising, as DEX is known to be a hygroscopic polymer which can retain water [50, 51]. Based on this, the more DEX is added, the more water is retained in the dried particle and the slower the evaporation rate, leading to the mentioned voluminous particles having a high AR. These described differences in droplet drying dynamics seem to destabilize the protein X formulation in the presence of high concentrations of DEX, confirmed by the observation that more aggregates were detected in these samples (Fig. 3). We hypothesized, the destabilization here is caused by phase separation of DEX and protein X during sessile droplet drying. The steric hindrance of large DEX molecule will not allow the two molecules to readily come close enough to build out hydrogen bonds [52]. For other polymers it has been observed, that increased solid concentrations of these type of excipients, led to phase separation and increased aggregation in the protein rich phase [53, 54]. Furthermore, this phase separation during sessile droplet drying, caused by DEX and other species of high molecular weight, has been elucidated recently [55]. In the latter work, the authors showed that DEX has a tendency to accumulate at the contact line between droplet and surface, whereas other species of high molecular weight, like polyethylene glycol, rather show a tendency to stay at the droplet centre [55]. We propose, that a similar phenomenon might be at work during our presented experiments.

Following these results and to gain more information on the relevance of these phenomena, we spray dried all discussed formulations of different S/P molar ratios. The results of these spray drying runs will be discussed in the next section.

### Spray Drying (SD)

In order to understand how these observations obtained during MD could translate to larger scale drying, during which the evaporation timescales are a great deal faster, the same S/P ratios have been used to prepare protein X formulations for spray drying experiments. The spray drying experiments should give us more details on the destabilization of HPβCD and the performance of the other two saccharides when drying protein X in larger scale in their presence and absence.

#### Impact of SD on Protein Stability During Drying

**Fig. 9:**
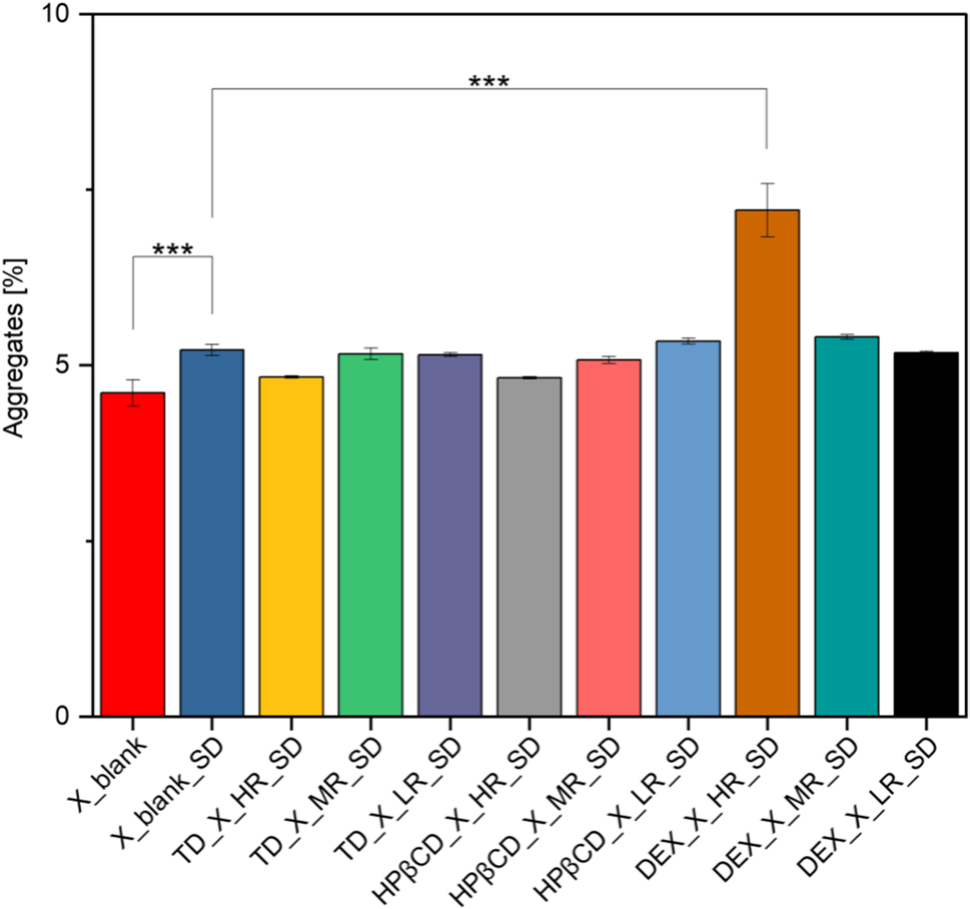
Statistical evaluation of the aggregates obtained by SEC-analysis (*n* = 3) of dried powders after SD (statistical differences are noted with asterisks: * *p* <  = 0.05, ** *p* <  = 0.01 and *** *p* <  = 0.001). The Bonferroni correction was applied, which is more suitable for a smaller sample size. Blank = ProtX formulation without saccharides, undried.

When protein X was spray dried without the presence of any saccharides in the formulation, a statistically significant increase in aggregates was observed, compared to the original lyophilized powder used here also (Fig. 9). The WAXS analysis *(n* = *3)* of all the different spray dried powders containing protein X, showed typical amorphous halo for all samples (supplementary Figure, S1).

The percentage of aggregation after SD in presence of saccharides at their HR, is presented (as mean ± standard error) in increasing order and compared to protein X without saccharides: TD (4.83 ± 0.01) < HPβCD (4.82 ± 0.01) < X (5.22 ± 0.04) < DEX (7.21 ± 0.31). Based on these observations it can be assumed, that protein X is best stabilized in terms of lowest aggregation using the disaccharide TD or the cyclic oligosaccharide HPβCD in the formulation. This means, that even small amounts of TD did not lead to a statistically significant increase in protein aggregation after SD and still were able to protect the protein’s structure. It can be attributed to the smaller size of this disaccharide and its higher molecular flexibility, due to which the miscibility between protein and saccharide is increased, and hence, protein stabilization is improved. Moreover, such smaller disaccharides are able to get into very close contact with the surface of the protein as they are less limited by steric hindrance, by which larger saccharides would be strongly affected. Because of steric hindrance, such larger saccharides cannot get in close contact with the surface of the protein and therefore, could lead to the formation of cavities causing destabilization of the protein [15, 56]. After all, it is generally known, that the strength of hydrogen bonds depends strongly on the distance between the two bonding partners, meaning, the closer the bonding partners (namely saccharide and protein in this scenario) can get into contact, the stronger the hydrogen bonds created. Clearly, steric hindrance would have a strong negative impact on the successful formation of hydrogen bonds. It is noteworthy, that for disaccharides like TD, which are naturally smaller in size than oligo-or polysaccharides, the mechanism of protein stabilization is most probably attributed to the formation of hydrogen bonds with the protein [57]. Concerning the main mechanisms of protein stabilization by TD, vitrification theory is the first one in which the protein is captured and restricted in its movements by the glassy matrix of TD. Secondly, the preferential exclusion or water replacement theory, in which the backbone of a protein could be targeted by TD-molecules for favourable binding. When in its unfolded (denatured) state, the protein’s surface area is much larger than it would be in its folded (native) state, providing more area for TD-molecules to bind to [58].

The spray dried protein X formulations containing HPβCD at its HR showed even a lower percentage of aggregation when compared to the protein X spray dried without any saccharides (Fig. 9). It is very interesting to observe that, as the content of HPβCD is reduced, the mean percentage of aggregation in the spray dried powders (± standard error) increases as follows: HR (4.82 ± 0.01) < MR (5.07 ± 0.04) < X (5.22 ± 0.04) < LR (5.34 ± 0.03). This observation is contradictory to the observations made with HPβCD in the MD. Milani *et al*., 2020, reported different ways in which HPβCD could stabilize proteins during drying as well as that this stabilization could be dependent on a molecular level. Furthermore, they report that HPβCD could stabilize proteins in two ways: either it might bind to hydrophobic residues of amino acids or it acts on the surface of the protein and protects the structure in this way, but it is difficult to pinpoint the relative contribution of these mechanisms [41].

Furthermore, in contrast to the findings and conclusions reported about HPβCD by Milani *et al*., 2020, Rospiccio *et al*., 2021 connected the mentioned destabilizing effect with the very weak surface activity of HPβCD [41, 59]. According to the authors, this saccharide will be inefficient in stabilizing a surface active protein during drying [59]. In our case, the protein X alone has shown a similar surface activity as HPβCD within the examined liquid formulations, namely 57.10 ± 2.08 mN/m for HPβCD at HR and 52.76 ± 0.63 mN/m for protein X. The authors further postulated the amphiphilic character as a hypothesis for stabilization success of HPβCD as the interaction takes place between its hydrophobic cavity and the backbone of the protein [59]. Ohtake *et al*., 2011, pointed out that during the atomization step in SD of proteins, the interfacial surface area between water and air is very large. Therefore, one of the mechanisms in which HPβCD could stabilize the protein, would be to compete with the water-air interface. On top of all, the one being reported to be the most important, once again is the inhibition of denaturation of proteins at the surface by surface active agents [60]. In this scenario, polysorbate 80 and HPβCD, can both be present on the surface.

All of these mentioned observations are congruent in our results, that HPβCD tends to destabilize the protein during SD and MD, but more prominent during MD, confirming our results about the non-translatability with this saccharide between the two drying approaches. The main protein stabilization by HPβCD is assumed to be caused by the mechanism of interfacial competition. The interfacial area available during MD is much smaller than for the droplet generated during SD for the same volume of liquid. Thus, this interfacial competition would be less relevant or at least not comparable between SD and MD. Especially for HR used during MD, the availability of only a smaller interfacial area might lead to destabilization of the protein by HPβCD. In contrast, the interfacial area available during SD is much larger and hence, HPβCD used at HR will be equally useful in protein stabilization as the MR and LR. Admittedly, the exact molecular mechanism behind the protein destabilization by HPβCD during evaporative drying could not be elucidated from the present data we have. In addition, despite there are published studies reporting HPβCD binding to nonpolar segment of protein in liquid formulation [40, 59], such data are rare or not existing to our knowledge and would be crucial for advancing HPβCD as the solid biologics excipient for spray dried products.

When looking at the performance of DEX, a statistically significant reduction of aggregates was observed after drying, when DEX was used at its LR in the formulations with protein X (Fig. 9). However, the percentage of aggregates increases with increasing DEX concentration. This observation is in agreement with that made during MD. Hence, DEX used in low amounts could therefore be a potential candidate in stabilization of protein structure during drying.

#### Impact of SD on Protein Particle Formation

Spray drying of protein X formulations without the presence of any saccharides (formulation X-blank_SD in Table III) yielded particles with a size of 12.10 ± 0.37 µm, which was found to be an intermediate size when comparing to the sizes of spray dried formulations with presence of saccharides (Table III). The addition of different saccharides to the protein X formulation indeed led to significant differences in the particle size span, SMD, VMD and S/V ratio, depending on the saccharides used. When TD was used in HR, the particle size increased, compared to protein X alone. However, the particle sizes tended to decrease, when TD was used in MR or LR in the formulations.

**Table III:** Results of PSD and KF Analysis of Spray Dried Powders are Summarized

Formulations	PSS	SMD / µm	VMD / µm	S/V ratio	Moisture content /%
X_blank_SD	1.60 ± 0.05	5.28 ± 0.31	12.10 ± 0.37	0.44 ± 0.02	7.38 ± 0.13
TD_X_HR_SD	1.70 ± 0.06	5.41 ± 0.25	13.03 ± 0.25	0.41 ± 0.01	6.59 ± 0.66
TD_X_MR_SD	1.83 ± 0.08	4.74 ± 0.25	11.36 ± 0.43	0.42 ± 0.01	5.19 ± 0.28
TD_X_LR_SD	1.79 ± 0.01	4.83 ± 0.07	10.85 ± 0.11	0.45 ± 0.00	5.10 ± 0.22
HPβCD_X_HR_SD	1.66 ± 0.02	5.22 ± 0.19	12.02 ± 0.28	0.43 ± 0.01	6.98 ± 0.15
HPβCD_X_MR_SD	1.88 ± 0.03	4.41 ± 0.15	10.05 ± 0.30	0.44 ± 0.01	4.69 ± 0.07
HPβCD_X_LR_SD	1.93 ± 0.02	4.11 ± 0.11	9.32 ± 0.32	0.44 ± 0.00	5.39 ± 0.21
DEX_X_HR_SD	2.82 ± 0.04	5.97 ± 0.10	21.05 ± 0.40	0.28 ± 0.00	8.02 ± 1.36
DEX_X_MR_SD	1.89 ± 0.06	5.47 ± 0.33	13.65 ± 0.56	0.40 ± 0.01	5.91 ± 1.59
DEX_X_LR_SD	1.82 ± 0.04	5.25 ± 0.09	12.32 ± 0.30	0.43 ± 0.00	7.39 ± 0.75

The mean values of PSS (Particle Size Spans), SMD (Sauter Mean Diameter), VMD (Volume Mean Diameter), S/V ratio (Surface-to-Volume ratio) and moisture content are presented for formulations of different S/P molar ratios. (Note: Values are presented as mean ± standard error, *n* = 3)

In the case of HPβCD, the particle sizes significantly change, when used in lower amounts within the formulation (Table III). Here, HPβCD used at HR leads to a particle size of 12.02 ± 0.28 µm, which is similar to the one observed for protein X without any saccharides. In more detail, the principle of atomization during spray drying is based on decreasing the surface tension of the liquid feed, to achieve formation of spherical droplets of small size, as the authors state [61]. This nearly 100-fold decrease in saccharide amount between HR and LR, leads to particles of size 9.32 ± 0.32 µm. Hence, for HPβCD the same clear trend is observed that using lower amounts in the formulations produces smaller particles (Table III). The surface tension of different formulations are provided in the supplementary information (Table S2). HPβCD is a special case as it might be able to cause molecular inclusion and act as a surfactant. Based on the results it seems, that there is an ideal molar ratio, in which it could be used [59, 62].

**Fig. 10:**
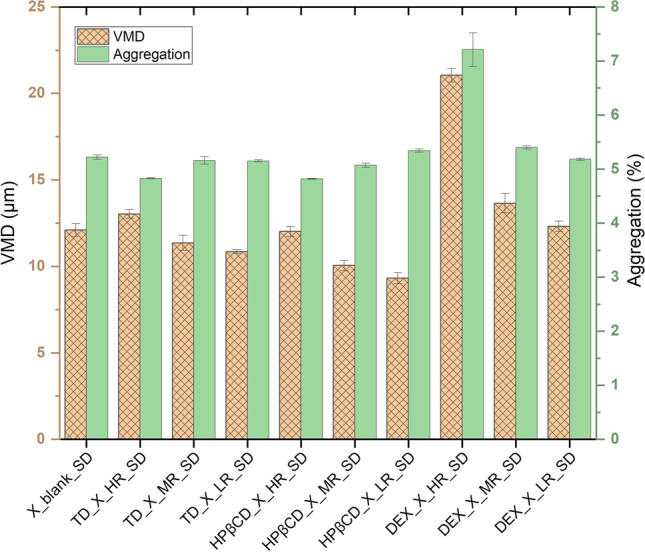
Comparison of particle sizes (µm) and aggregation of different spray dried formulations. Presented values are mean values and corresponding standard deviations are shown as error bars.

Moving further to DEX it was observed, that using DEX in the formulations increased the particle size in general. The higher the DEX content, the smaller the S/V ratio, the higher the aggregation (Fig. 10) and the larger the particle size with 21.05 ± 0.40 µm presenting the largest particle size of all examined formulations (Table III).

Comparing DEX used in its HR against its LR, a decrease in particle size of around 40% can be observed. This nearly tenfold decrease in saccharide amount between HR and LR, leads to particles with a size of 12.32 ± 0.30 µm. Mandato *et al*., 2012, reported that the droplet size generated by a nozzle is larger, if the density, surface tension or the viscosity of a formulation is increased [63]. The largest particles were obtained with DEX with a size of 21.05 ± 0.40 µm, compared to all other formulations tested, after SD. Underlining what we have observed, the authors mention, that a highly viscous solution is expected to increase its resistance to the force of atomization, which will lead to increased particle sizes. Although for bi-fluid nozzles, the influence of the hydrodynamic properties of the formulations are not as strong as for other nozzle types, for example mono-disperse droplet generators, during this work, the impact of these properties could be detected when DEX was used in its HR [63]. Ohtake *et al*., 2011, reported for lyophilization, that DEX of certain molecular weights (for example DEX of 40 kDa size) fail to stabilize proteins most probably due to their size, causing steric hindrance [60], which has already been explained in Sect. 3.1.2. Amongst other important interactions, strong hydrogen bonds are essential for a stable secondary protein structure [52]. Additionally, the moisture content for spray dried protein formulations containing DEX, is the highest compared with other saccharides used (Table III). It might be that more time or seconday drying is required for lowering the moisture content, as these droplets are larger in size and DEX is hygroscopic. Furthermore, the moisture content with DEX shows similar values as for the formulation, in which no saccharide was present and protein alone was dried. Furthermore, the more DEX used in the formulations, the higher the moisture content of the spray dried powder and the slower the previously discussed evaporation rates in MD. This suggests to use as less DEX as possible in the formulation, if smaller, drier particles are desired.

### Comparison of Particle Behaviour and In-Process Stability of MD *Versus* SD

The S/V ratios presented in Fig. 7 (for MD) and in Table III (for SD) support the statement that particles produced by SD are generally of much smaller sizes than particles produced by MD.

**Fig. 11:**
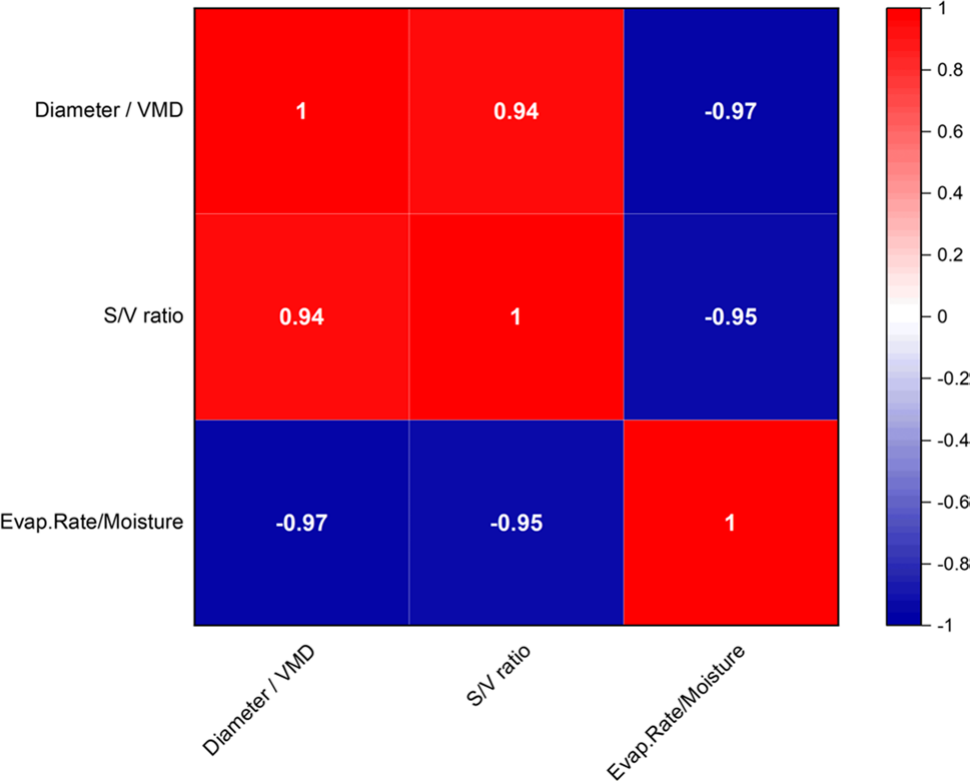
The Pearson correlation plot is presented. Equivalent data obtained from MD and SD are analysed to detect any possible correlations between the two techniques. The numbers indicate the nature of correlation, namely a negative correlation for values around -1, a positive correlation for values around + 1 and the value 0 indicates no correlation.

The Pearson’s r correlation plot (Fig. 11) shows strong linear (significant) connections between the chosen parameters for MD and SD experiments as their correlation parameters are very close to + 1 and/or -1. Negative linear (significant) correlations (deep blue colours) are observed between “diameter/VMD” with “evaporation rate/moisture” and “S/V ratio” with “evaporation rate/moisture”.

Furthermore, positive linear (significant) correlations (deep red colours) are observed between “diameter/VMD” with “S/V ratio”. This means, naturally, that the evaporation rate and the moisture content will decrease as the droplet diameters decrease. The correlation coefficients were -0.97 (*n* = 20, *p*-value < 0.0001, 95% CI) and -0.95 (*n* = 20, *p*-value < 0.0001, 95% CI).

Moreover, the S/V ratio of the droplets will increase as their diameters increase. Here, the correlation coefficient was 0.94 (*n* = 20, *p*-value < 0.0001, 95% CI). In summary, this correlation proves the comparability of the results of the presented two drying techniques as it represents a highly significant connection. It shall be mentioned that for this correlation, it neither can be accounted for possible shear forces being present during SD and absent during MD, nor interfacial phenomena like the Marangoni effect being more pronounced during MD than SD.

**Fig. 12:**
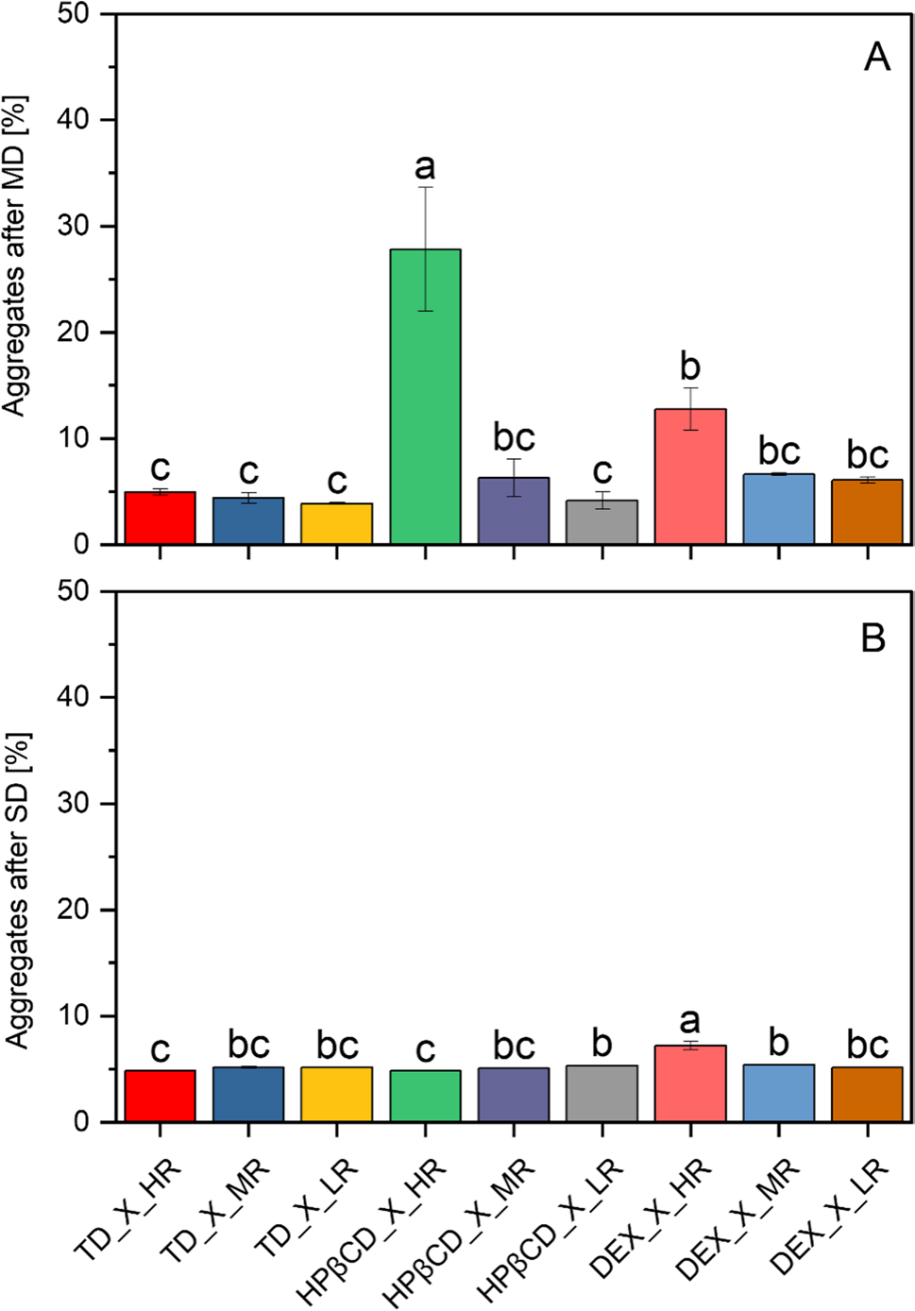
Aggregates obtained by SEC analysis of droplets by MD (**A**) and powders by SD ﻿(**B**) of different S/P molar ratios has been statistically compared (pairwise). Similar performance of excipients is shown by automatic categorizing into corresponding groups a, b and c. Presented values are mean values and corresponding standard deviations are shown as error bars. The Bonferroni correction was applied, which is more suitable for a smaller sample size. A significance level of 0.05 was used.

In the case of HPβCD, the results of SD and MD are not entirely comparable (Fig. 12) and further experiments need to be carried out to gain deeper knowledge about the (de)stabilization mechanism of this saccharide. Lower amounts of saccharides actually work for SD and for MD, but the same is not true for higher amounts, which do not work for MD and show tremendously increased aggregation (group “a” in Fig. 12A). After a statistical pairwise comparison of aggregation between MD (Fig. 12A) and SD (Fig. 12B), it is obvious that HPβCD at HR after MD behaves different than all other ratios. Therefore, it has been categorized into a different group than the other HPβCD ratios. Although DEX at HR stands out as well in group b, it, however, is grouped within the same groups as the other DEX ratios used during MD. In the case of SD, all saccharides are categorized into similar groups, except DEX used at HR, which was sorted into its own group.

In this work it has been shown that different M_w_-saccharides lead to different droplet sizes. For the MD approach, larger particles have been produced. For SD, in contrast, drying happened much faster and smaller droplets have been produced in general. Comparing the MD with a “pinned” droplet approach and the SD experiments with “non-pinned” or contactless droplet drying approach, a difference in solute movement during evaporation can be expected. During evaporation and drying of a droplet, the droplet shrinks and the solute will be enriched at the droplet surface, at which the solvent is being evaporated [38]. Saccharides have smaller molecular weights than the larger proteins, which are therefore much slower in diffusing away from the droplet’s surface to the droplet’s centre during drying, making them prone to aggregation at the interface [38, 64].

## Conclusion

Our study made a direct comparison of different saccharides used at varying ratios as the stabilizers of protein during a miniaturized single droplet drying (MD) and spray drying (SD). During the MD, HPβCD and DEX used at high ratio (HR) were not able to protect the protein against aggregation, compared with other ratios and saccharides and hence led to the destabilization of the protein. In contrast, during SD, similar results were observed for DEX but not for HPβCD. While the developed MD approach was able to anticipate the in-process protein stability of formulations containing protein X and the saccharides TD or DEX, it yielded contrasting results for using HPβCD. Due to the obvious differences in the drying configuration of two techniques and different possible mechanisms involved in the in-process (de-) stabilization by the selected excipients, protein formulation screening based on miniaturized single droplet drying, might not always be representative of SD outcomes. For instance, the non-translatability of MD to SD in the case of HPβCD could be due to the discussed higher air–liquid ratio offering a larger interface with more HPβCD molecules being present during SD. However, the differences observed between the two set-ups, underline that the use of different saccharides and respective ratios need to be carefully considered, depending on the drying unit operation to be used. As was seen in this work, the stabilization mechanism can strongly depend on the drying principle, when comparing the results for HPβCD between MD and SD.

Based on these observations using protein X, further experiments are required to translate this MD approach to other protein therapeutics and for the specifically with the contrasting results given by HPβCD. At present, we are carrying out succeeding studies using identical approach for the known proteins presenting distinct molecular weights and chemistry. We also further hope to get more insights into the (de- stabilization mechanism of distinct saccharides at different scales (MD and SD) through molecular modeling. Additionally, a wider space of SD process parameters need to be explored. Altogether, learnings and available information on the excipient selection for protein SD can be better understood and will no longer be only based on the field of lyophilization of biologics.

## Supplementary Information

Below is the link to the electronic supplementary material.

Supplementary file1 (DOCX 445 KB)

## Data Availability

Data are available upon request.
