# Supplementary material for: Impact of Different Saccharides on the In-Process Stability of a Protein Drug During Evaporative Drying: From Sessile Droplet Drying to Lab-Scale Spray Drying
Source: Pharm Res. 2023 Apr 3;40(5):1283–98. doi: 10.1007/s11095-023-03498-w (PMC10229717; doi:10.1007/s11095-023-03498-w)
Supplement: Supplementary file 1 — Supplementary file1 (DOCX 445 KB) [file 11095_2023_3498_MOESM1_ESM.docx]

# Supplemental material

The following supporting figure S1 represents the amorphous character of all spray dried samples:


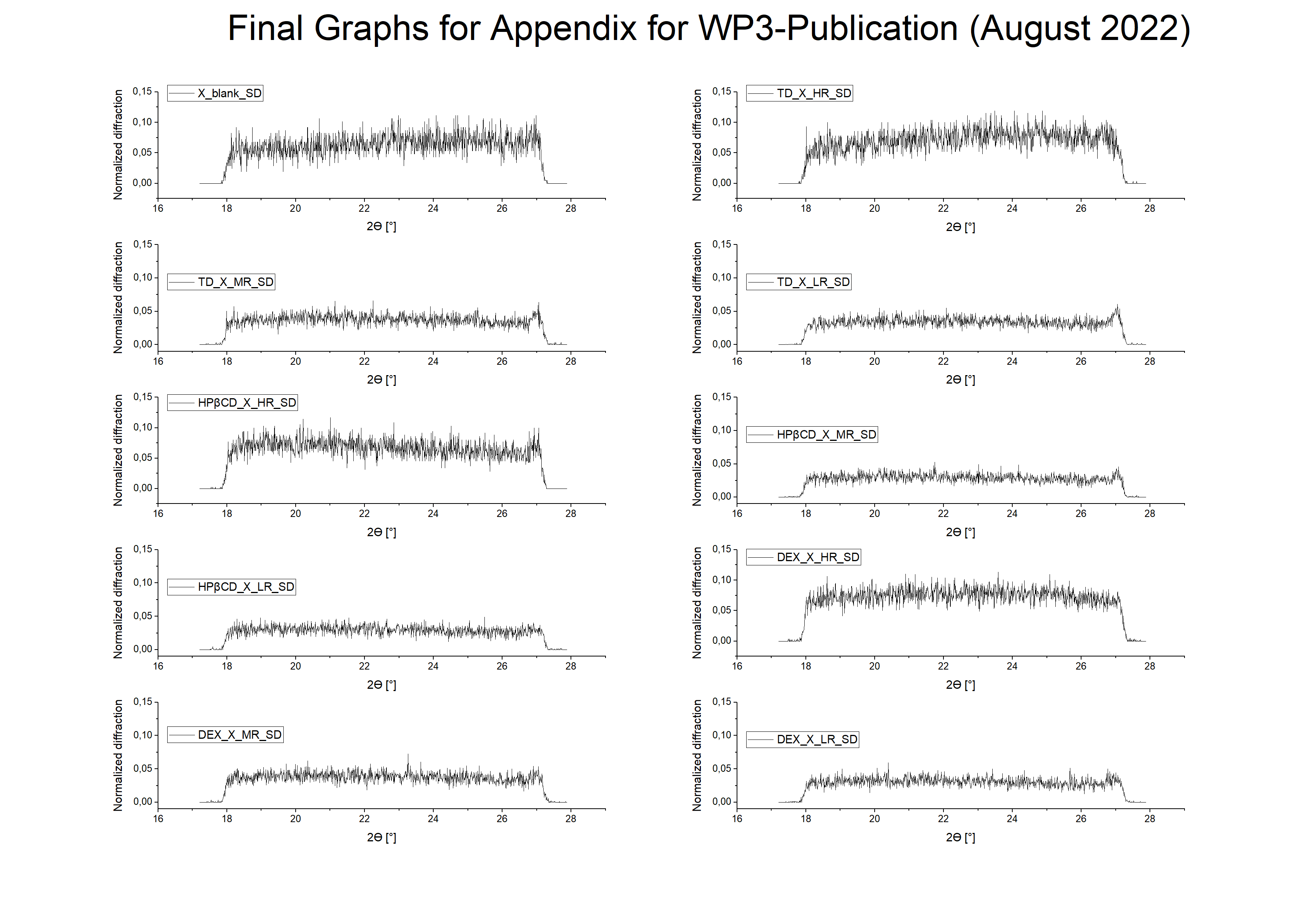


**Figure S1:** WAXS spectra for representation of the amorphous character of all spray dried samples produced in this work.

The following supporting information describes the surface tension methodology and respective results (Table S3):

**Surface Tension Measurements:**

First, as required for following surface tension measurements, the densities [g/cm3] of the different S/P formulations were measured on a DSA 5000 M density and sound velocity meter (Anton Paar GmbH, Austria). After obtaining the densities of the formulations, the surface tension was measured on the mentioned EasyDrop equipment (EasyDrop, Krüss GmbH, Germany) with the software Drop Shape Analysis (DSA1 v1.92, Krüss GmbH, Germany). Using the provided syringe, a single droplet with a volume as large as possible was dispensed without tearing off the syringe tip. Of six single droplets from each formulation, the interfacial tension [mN/m] was determined and summarized in Table S2.

Table S2: Results of the surface tension measurements of different solutions containing only one saccharide (no protein present) in water at HR are summarized. The mean values are presented. (Note: Values are presented as mean ± standard error, n=5).

| **Formulations** | **Surface Tension (Interfacial tension, IFT) / mN/m** |
| --- | --- |
| X_blank | 52.76 ± 0.63 |
| TD_HR | 71.51 ± 0.63 |
| HPβCD_HR | 57.10 ± 2.08 |
| DEX_HR | 70.50 ± 1.64 |
